# Pattern of coronary arterial lesions amongst Saudi Arabians: a cross-sectional coronary fluoroscopic angiography study

**DOI:** 10.11604/pamj.2020.36.21.21423

**Published:** 2020-05-18

**Authors:** Khalid Hadi Aldosari, Khalid Mansour Alkhathlan, Sameer Al-Ghamdi, Fayez Elsayed Abdelhamid Elshaer, Mohammed Hamid Karrar, Abdulrahman Mohammed Aldawsari

**Affiliations:** 1 Prince Sattam Bin Abdulaziz University, Colleges of Medicine, Al-Kharj, Saudi Arabia; 2 Department of Family Medicine, College of Medicine, Prince Sattam Bin Abdulaziz University, Al-Kharj 11942, Saudi Arabia; 3 King Khaled University Hospital, King Fahad Cardiac Center, King Saud University, Riyadh, Saudi Arabia; 4 National Heart Institute, Cairo, Egypt; 5 Department of Basic Medical Sciences, Colleges of Medicine, Prince Sattam Bin Abdulaziz University, Al-Kharj 11942, Saudi Arabia

**Keywords:** Coronary artery disease, cardiovascular disease, Saudi Arabia

## Abstract

**Introduction:**

Coronary artery disease (CAD) is a major cardiovascular disease (CVD) that affects a large population globally. This study aimed at determining coronary arterial lesions (CAL), particularly in terms of age, gender, coronary artery/arteries involved, number of lesions, and dominant coronary artery in the Kingdom of Saudi Arabia (KSA).

**Methods:**

A cross-sectional study was conducted at the King Khalid Hospital and Prince Sultan Centre for Health Care in Al-Kharj between January 2017 and March 2018. The patients with CAD lesion/s, fulfilling the inclusion criteria, were recruited from the cardiovascular medicine unit. Demographic information and the location and extent of their CAD lesions were extracted and documented in electronic case report form (eCRF). SPSS 22.0 was used for statistical analysis, and p value ≤ 0.05 was considered as significant.

**Results:**

Of the 262 patients, male and female preponderance was 74.8% and 25.2%, respectively. The majority of the patients were adults above the age of 50 (72%). About half of all patients were active smokers (53%). Diabetes, hypertension, and hyperlipidaemia were recorded in 63%, 53.7% and 25% respectively. The incidence of cardiovascular lesions was documented after coronary angiography; left circumflex artery lesions had the highest incidence (85.3%), followed by left anterior descending artery lesions (82.4%) and right circumflex artery lesions (74.3%). Left main coronary artery lesions had the lowest incidence (10.3%). Most patients (59.6%) had three concomitant lesions, whereas a minority of patients had two (22.8%) and one lesion (17.7%).

**Conclusion:**

The pattern of CALs is different among the Saudi population as compared to other countries.

## Introduction

Coronary artery disease (CAD) is a major cardiovascular disease (CVD) that affects approximately 422.7 million people globally [1, 2]. In the United States alone, 15.5 million individuals above 20 years of age suffer from coronary heart disease (CHD) [3]. Globally, 17.5 million people died of cardiovascular disease (CVD) in 2012; 7.4 million deaths were contributed to CAD [4]. Prevalence of CAD in the Kingdom of Saudi Arabia (KSA) has been reported to be 6.4% of men and 4.4% of women [5]. According to the statistics of World Health Organization (WHO) 2016, about 37% of deaths were attributed to CVD [6], indicating KSA bears a significant burden of CAD, like other countries in the world. Increasing age is a well-established risk factor for CAD. For instance, incidence of CHD is doubled and tripled for men and women, respectively, at 65-94 years as compared to that at 35-64 years [3]. The reason for this increased risk of CAD can be attributed to the progressive decline of physiological functions, which leads to health problems. Similarly, increasing age affects the cardiovascular system in terms of pathological alterations (e.g. hypertrophy, left ventricular dysfunction, arterial stiffness, impaired endothelial function, etc.), contributing to atherosclerosis, hypertension, and myocardial infarction (MI) [7]. As incidence of hypertension increases with advancing age, it further increases the risk of CAD.

Gender differences at the same age exist for the incidence of CAD. Men are at higher risk of developing CAD as compared to women of the same age [8]. Lifetime risk of developing CHD for men and women aged 40 years is 49% and 32% respectively, while it is 35% for men and 24% for women at age 70 years [3]. Similarly, women lag behind men by 10-20 years in terms of occurrence of coronary events [3]. There might be various reasons for these gender differences in the development of CAD. For example, women are likely to have non-obstructive CAD and take longer to obtain medical advice as compared to men [9]. In KSA, studies are lacking on the different lesions of CAD. In this study, we aimed at determining coronary arterial lesions (CAL), particularly in terms of age, gender, coronary artery/arteries involved, number of lesions, and dominant coronary artery in KSA. This study is a valuable addition to the literature on arterial lesions of CAD in KSA.

## Methods

This topic was chosen for in-depth research to fulfill the aim of determining the prevalence and pattern of coronary artery disease (CAD) lesions amongst cardiovascular patients at a tertiary healthcare institution in the Kingdom of Saudi Arabia (KSA). Demographic variables, such as age and gender, were analyzed to identify potential associations among variables and prevalence of CAD. A comprehensive search of the literature was undertaken on medical databases (e.g. PubMed, Embase and Medline) with the following search terms - KSA, Saudi, coronary, ischemic, lesion, artery. Boolean operators, such as AND and OR, were utilized to generate productive and focused results. The search produced studies that explored the prevalence of CAD in KSA. However, to the best of the authors´ knowledge, no study has been scoped to determine the prevalence of specific CAD lesions amongst the KSA population. Hence, a cross-sectional study set out to delineate this phenomenon was planned and conducted between January 2017 and March 2018. This cross-sectional study was conducted in the King Khalid Hospital and Prince Sultan Centre for Health Care in Al-Kharj; 262 patients were recruited and subjected to stringent inclusion and exclusion criteria. Patients were recruited from the cardiovascular medicine (CVM) unit. The inclusion criteria for the study were as follows: male and female genders, all ages, provision of informed consent by the patient or a legally appointed guardian, residence within KSA, presented to the King Khalid Hospital and Prince Sultan Centre for Health Care, admitted to the CVM unit for whatever reason, indicated and completed a coronary angiogram, and at least one documented CAD lesion on said coronary angiogram. The exclusion criteria for this study were as follows: minors who failed to provide legal guardian consent, patients who had deceased upon admission to the CVM unit, patients who had deceased after admission to the CVM unit and before obtaining a complete coronary angiogram, patients who were indicated for other forms of coronary artery imaging (e.g. magnetic resonance imaging, multislice computed tomography and electron beam computed tomography), and patients whose coronary angiogram revealed no CAD lesions. All 262 patients who were recruited into the study were identified at the point of their admission into the CVM unit. Informed consent, according to Good Clinical Practice (GCP) guidelines was obtained, and demographic information was extracted from patients´ electronic health records. This information was documented in the electronic case report form (eCRF). Similarly, the location and extent of their CAD lesions were extracted and documented in the eCRF. This quantitative data was analysed anonymously by the investigators. SPSS 22.0 was used for statistical analysis, and the threshold for significance was deemed to be at least 0.05.

## Results

**Figure 1:**
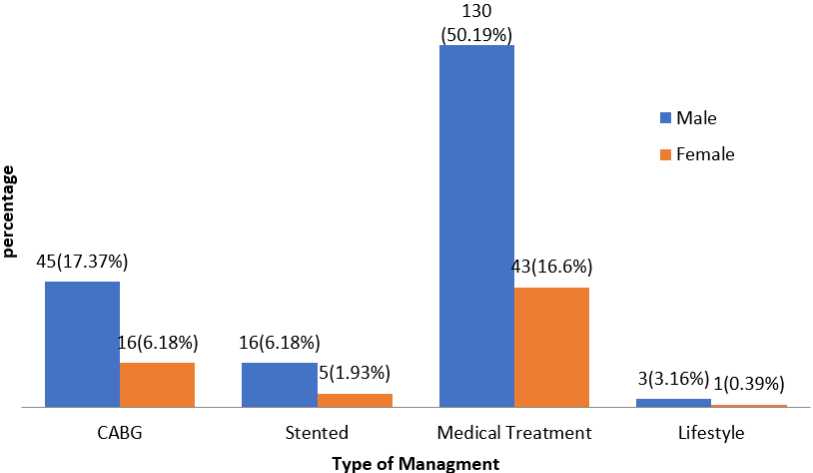
Management of CAD

**Figure 2:**
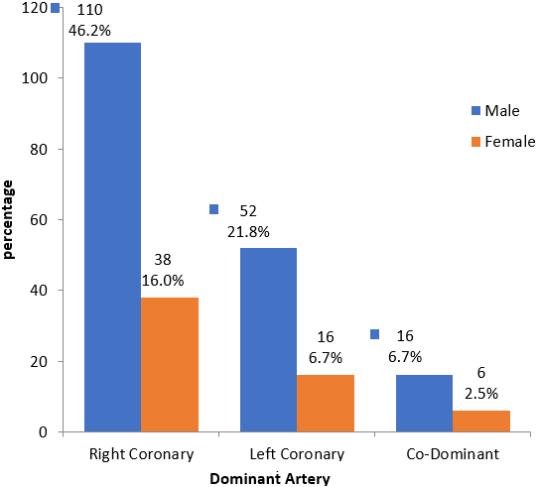
Prevalence of dominant coronary artery

The aim of this study was to delineate the lesions of coronary artery disease (CAD) according to age and gender amongst a patient pool in the Kingdom of Saudi Arabia (KSA). Of the 262 patients recruited for this study, there was an overall male preponderance (74.8%) compared to females (25.2%). The majority of these patients were adults above the age of 50 (72%). About half of all patients were active smokers (53%). In regard to comorbid disease, 25% of patients had documented hyperlipidaemia, 53.7% had chronic hypertension, and 63% were chronic diabetics. The incidence of cardiovascular lesions was documented after coronary angiography - left circumflex artery lesions had the highest incidence (85.3%), followed by left anterior descending artery lesions (82.4%) and right circumflex artery lesions (74.3%). Left main coronary artery lesions had the lowest incidence (10.3%). Most patients (59.6%) had three concomitant lesions, whereas a minority of patients had two (22.8%) and one lesion (17.7%). These figures can be referenced in detail in Table 1. Associations between the lesions of coronary artery disease and demographic indices were observed. For example, a statistically significant (p = 0.001) association between the presence of a left circumflex artery lesion and age was found. Specifically, as age increased, there was a higher likelihood of a left circumflex artery lesion, as delineated in Table 2. Similarly, there was a statistically significant (p = 0.026) association between the presence of a right coronary artery lesion and age. However, these associations should be interpreted with caution; the sample size of patients in specific age categories was between 0 and 5 in more than 20% of all age categories. A larger sample size would have lent more credibility to the associations observed. There were no statistically significant associations observed between age and the presence of left main coronary artery or left anterior descending artery lesions. There was also no statistically significant association between age and the number of affected vessels. Age was not associated with the type of management (coronary artery bypass grafting, endovascular stenting, or conservative treatment). The distribution of management strategies is reflected in Figure 1. No statistically significant associations were observed between gender and CAD lesions, as reflected in Table 3. There was also no statistically significant association observed between the dominant coronary artery and gender. The prevalence of the dominant coronary artery amongst the patient pool is reflected in Figure 2.*’*

**Table 1:** Demographic data and coronary artery disease (CAD) lesions

		Frequency (%)
**Gender**	Male	196 (74.81%)
	Female	66 (25.19%)
**Age**	Less than 30	6 (2.21%)
	between 30 to 40	16 (5.88%)
	between 40 to 50	54 (19.85%)
	between 50 to 60	88 (32.35%)
	More than 60	108 (39.71%)
**Smoking status**	Yes	142 (52.99%)
	No	126 (47.01%)
**Dyslipidemia**	Yes	68 (25%)
	No	204 (75%)
**HTN**	Hypertensive	146 (53.68%)
	Non-hypertensive	126 (46.32%)
**Diabetes**	Diabetic	170 (62.96%)
	Non-diabetic	100 (37.04%)
**LM lesion**	Yes	28 (10.29%)
	No	244 (89.71%)
**LAD Lesion**	Yes	224 (82.35%)
	No	48 (17.65%)
**LCX Lesion**	Yes	232 (85.29%)
	No	40 (14.71%)
**RCA lesion**	Yes	202 (74.26%)
	No	70 (25.74%)
**No. of lesions**	Single artery lesion	48 (17.65%)
	Two arteries lesion	62 (22.79%)
	Three arteries lesion	162 (59.56%)
**Dominant Artery**	Right Coronary A	150 (61.48%)
	Left Coronary A	72 (29.51%)
	Co-dominant	22 (9.02%)

**Table 2:** Associations between age and CAD lesions

		Less than 30	between 30 to 40	between 40 to 50	between 50 to 60	More than 60	Age P value
LM lesion	Yes	0 (0%)	0 (0%)	6 (2.21%)	12 (4.41%)	10 (3.68%)	0.440^a^,^b^
	No	6 (2.21%)	16 (5.88%)	48 (17.65%)	76 (27.94%)	98 (36.03%)	
LAD Lesion	Yes	6 (2.21%)	10 (3.68%)	46 (16.91%)	76 (27.94%)	86 (31.62%)	0.114^a^
	No	0 (0%)	6 (2.21%)	8 (2.94%)	12 (4.41%)	22 (8.09%)	
LCX Lesion	Yes	2 (0.74%)	12 (4.41%)	44 (16.18%)	74 (27.21%)	100 (36.76%)	0.001^a^,^b^,*
	No	4 (1.47%)	4 (1.47%)	10 (3.68%)	14 (5.15%)	8 (2.94%)	
RCA lesion	Yes	4 (1.47%)	14 (5.15%)	48 (17.65%)	64 (23.53%)	72 (26.47%)	0.026^a^,*
	No	2 (0.74%)	2 (0.74%)	6 (2.21%)	24 (8.82%)	36 (13.24%)	
No. of lesions	Single artery lesion	2 (0.74%)	4 (1.47%)	8 (2.94%)	16 (5.88%)	18 (6.62%)	0.515^a^
	Two arteries lesion	2 (0.74%)	4 (1.47%)	8 (2.94%)	18 (6.62%)	30 (11.03%)	
	Three arteries lesion	2 (0.74%)	8 (2.94%)	38 (13.97%)	54 (19.85%)	60 (22.06%)	
Management	CABG	2 (0.74%)	2 (0.74%)	11 (4.09%)	19 (7.06%)	30 (11.15%)	0.708^a^,^b^
	Stented	1 (0.37%)	0 (0%)	6 (2.23%)	8 (2.97%)	6 (2.23%)	
	Medical Treatment	3 (1.12%)	14 (5.2%)	34 (12.64%)	59 (21.93%)	69 (25.65%)	
	Life>	0 (0%)	0 (0%)	2 (0.74%)	1 (0.37%)	2 (0.74%)	

Results are based on nonempty rows and columns in each innermost table.

* The Chi-square statistic is significant at the 0.05 level.

a More than 20% of cells in this subtable have expected cell counts less than 5. Chi-square results may be invalid.

b The minimum expected cell count in this subtable is less than one. Chi-square results may be invalid.

**Table 3:** Gender and CAD lesions

		Gender		
		Male	Female	Gender P value
LM lesion	Yes	20 (7.63%)	8 (3.05%)	0.663
	No	176 (67.18%)	58 (22.14%)	
LAD Lesion	Yes	164 (62.6%)	52 (19.85%)	0.367
	No	32 (12.21%)	14 (5.34%)	
LCX Lesion	Yes	168 (64.12%)	56 (21.37%)	0.863
	No	28 (10.69%)	10 (3.82%)	
RCA lesion	Yes	148 (56.49%)	50 (19.08%)	0.968
	No	48 (18.32%)	16 (6.11%)	
No. of lesions	Single artery lesion	34 (12.98%)	10 (3.82%)	0.996a
	Two arteries lesion	40 (15.27%)	20 (7.63%)	
	Three arteries lesion	122 (46.56%)	36 (13.74%)	
Dominant Artery				0.254a

Results are based on nonempty rows and columns in each innermost subtable. *. The Chi-square statistic is significant at the .05 level.

## Discussion

This cross-sectional study, which aimed at determining the prevalence of CAD lesions among the Saudi population, reported highest incidence of left circumflex artery (LCX) lesions and male predominance among cardiovascular patients. A significant association between the age of the patient and the involvement of LCX and right coronary artery (RCA) is also reported. Diabetes was reported to be the top risk factor for cardiovascular lesions. Moreover, the study revealed no association of age with left main (LM) coronary artery or LAD artery lesions or number of affected vessels. This unique study found that LCX is the most frequently involved coronary artery lesion involved in CAD. In this regard, the results of the present study are different from most of the previously performed studies, where other coronary arterial lesions have been reported to be most commonly involved, e.g. left anterior descending (LAD) artery. Ghanim *et al*. [10] reviewed 189 coronary angiograms of patients from Israel, who presented with ST segment elevation myocardial infarction (STEMI) with ≥50% arterial narrowing and reported LAD was the most prevalent coronary arterial lesion (36%-38%) among patients with STEMI, followed by RCA and LCX lesions (27 - 29%). The reason for this difference may be due to inclusion of only patients with STEMI and ≥50% narrowing of the arteries. However, they also demonstrated that LCX lesions found in patients with STEMI are even lower than those in total CAD. In total CAD, LAD, RCA and LCX are involved in 47%, 34% and 27% respectively, showing that LCX is found less in STEMI and collectively more when considering other forms of CAD, such as non-ST segment elevation myocardial infarction (NSTEMI) and unstable angina. This also indicates that involvement of LCX in CAD among the Saudi population required further evaluation in terms of risk factors, lesion characteristics, and management strategies. Similarly in China, Wang *et al*. [11] studied 5288 patients with CHD and assessed the lesions with coronary angiography. They reported maximum lesions of LAD followed by RCA and LCX, strengthening the point that arterial lesions among the Saudi population are different from other regions of the world. However, this difference can be attributed to the age ≤40 years, different cultural customs, or social norms among nations.

The present study revealed significant association between the age of the patient and the involvement of LCX and RCA. In Saudi Arabia, coronary arterial lesions (CALs) are significantly higher in the older population compared to younger people. However, this finding cannot be generalized, as the sample size of patients in specific age categories was between 0 and 5 in more than 20% of all age categories. Therefore, a larger sample size would lend credibility to the associations observed. In contrast, Tsai *et al*. [12] conducted a study in Taiwan, including 245 patients below 40 years of age suffering from acute coronary syndrome (ACS) and occlusive CAD. They reported maximum lesions in LAD, followed by LCX and RCA. Again, this study from Taiwan differed in terms of arterial lesions in CAD. Similarly, frequency of involvement of RCA lesion significantly differs with age. The present study revealed diabetes is the most common risk factor of CAD among the Saudi population, as 07 million people are diabetic and about 03 million people are pre-diabetic in Saudi Arabia [13]. Frequency of risk factors of CAD varies with geographical areas and advancing age. Nadeem *et al*. [14] conducted a study in Pakistan including 109 patients below the age of 45. They reported cigarette smoking (46%) as the most common risk factor of CAD, followed by family history (43%), high blood pressure (37%), dyslipidemia (33%), and diabetes mellitus (18%). These results indicate a huge difference in risk factors among the Pakistani and Saudi populations. This difference may be caused by age, below 45 years. In this regard, Abbot *et al*. [15] demonstrated that risk factors of CAD change with advancing age.

## Conclusion

This is the first comprehensive study on CALs from Saudi Arabia that reports the frequency of CALs in CAD among the Saudi population. It has revealed an important finding that the pattern of CALs is different among the Saudi population as compared to other countries. This difference may affect preventive and management strategies in KSA. Therefore, further evaluation through prospective studies on a large scale is necessary to find reasons for the different pattern of CALs in KSA.

### What is known about this topic

- Coronary artery disease is a major cardiovascular disease that affects approximately 422.7 million people globally;
- Prevalence of coronary artery disease in the Kingdom of Saudi Arabia has been reported to be 6.4% of men and 4.4% of women;
- In Saudi Arabia, approximately 37% of deaths are attributed to cardiovascular disease.

### What this study adds

- This is the first comprehensive study on coronary artery lesions from Saudi Arabia that reports the frequency of coronary artery lesions in coronary artery disease among the Saudi population;
- Left circumflex artery is the most frequently involved coronary artery lesion involved in coronary artery disease in Saudi Arabia;
- The pattern of coronary artery lesion is different among the Saudi population as compared to other countries.

## Competing interests

The authors declare no competing interests.

## References

[1] Roth GA, Johnson C, Abajobir A, Abd-Allah F, Abera SF, Abyu G (2017). Global, Regional, and National Burden of Cardiovascular Diseases for 10 Causes, 1990 to 2015. J Am Coll Cardiol.

[2] Benjamin EJ, Blaha MJ, Chiuve SE, Cushman M, Das SR, Deo R (2017). Heart disease and stroke statistics-2017 update. A report from the American Heart Association. Circulation.

[3] Sanchis-Gomar F, Perez-Quilis C, Leischik R, Lucia A (2016). Epidemiology of coronary heart disease and acute coronary syndrome. Ann Transl Med.

[4] Mendis S (2017). Global progress in prevention of cardiovascular disease. Cardiovasc Diagn Ther.

[5] Al-Nozha MM, Arafah MR, Al-Mazrou YY, Al-Maatouq MA, Khan NB, Khalil MZ (2004). Coronary artery disease in Saudi Arabia. Saudi Med J.

[6] World Health Organization (2018). Noncommunicable diseases (NCD) country profiles.

[7] North BJ, Sinclair DA (2012). The intersection between aging and cardiovascular disease. Circ Res.

[8] Pathak LA, Shirodkar S, Ruparelia R, Rajebahadur J (2017). Coronary artery disease in women. Indian Heart J.

[9] Regitz-Zagrosek V, Oertelt-Prigione S, Prescott E, Franconi F, Gerdts E, The EUGenMed Cardiovascular Clinical Study Group (2016). Gender in cardiovascular diseases: impact on clinical manifestations, management, and outcomes. European Heart J.

[10] Ghanim D, Kusniec F, Kinany W, Qarawani D, Meerkin D, Taha K (2017). Left circumflex coronary artery as the culprit vessel in ST-segment-elevation myocardial infarction. Tex Heart Inst .J.

[11] Wang X, Gao M, Zhou S, Wang J, Liu F, Tian F (2017). Trend in young coronary artery disease in China from 2010 to 2014: a retrospective study of young patients ≤45. BMC Cardiovasc Disord.

[12] Tsai WC, Wu KY, Lin GM, Chen SJ, Lin WS, Yang SP (2017). Clinical Characteristics of patients less than forty years old with coronary artery disease in Taiwan: a cross-sectional study. Acta Cardiol Sin.

[13] Al Dawish MA, Robert AA, Braham R, Al Hayek AA, Al Saeed A, Ahmed RA (2016). Diabetes mellitus in saudiarabia: a review of the recent literature. Curr Diabetes Re.

[14] Nadeem M, Ahmed SS, Mansoor S, Farooq S (2013). Risk factors for coronary heart disease in patients below 45 years of age. Pak J Med Sci.

[15] Abbott RD, Curb JD, Rodriguez BL, Masaki KH, Yano K, Schatz IJ (2002). Age-related changes in risk factor effects on the incidence of coronary heart disease. Ann Epidemiol.

